# Insulin resistance, endothelial function, angiogenic factors and clinical outcome in non-diabetic patients with chest pain without myocardial perfusion defects

**DOI:** 10.1186/s12933-016-0353-1

**Published:** 2016-02-19

**Authors:** Helena U. Westergren, Sara Svedlund, Remi A. Momo, Juuso I. Blomster, Karin Wåhlander, Erika Rehnström, Peter J. Greasley, Regina Fritsche-Danielson, Jan Oscarsson, Li-Ming Gan

**Affiliations:** Department of Molecular and Clinical Medicine, Institute of Medicine, Sahlgrenska Academy at the University of Gothenburg, Gothenburg, Sweden; Department of Clinical Physiology, Sahlgrenska University Hospital, Gothenburg, Sweden; AstraZeneca R&D, Gothenburg, Sweden

**Keywords:** Non-obstructive coronary artery disease, Insulin resistance, Outcome, Endothelial function, Growth factors

## Abstract

**Background:**

Patients with angina-like symptoms without myocardial perfusion scintigram (MPS)-verified abnormality may still be at risk for cardiovascular events. We hypothesized that insulin resistance could play a role in this population even without diagnosed diabetes. We further explored physiological and blood biomarkers, as well as global gene expression patterns that could be closely related to impaired glucose homeostasis to deepen our mechanistic understanding.

**Methods:**

A total of 365 non-diabetic patients with suspected myocardial ischemia referred to MPS were enrolled and followed up regarding event-free survival with a median time of 5.1 years. All patients underwent endothelial function assessment by reactive hyperemic index (RHI) using EndoPAT and extensive biomarker analysis. Whole blood global gene expression pathway analysis was performed in a subset of patients.

**Results:**

Homeostasis model assessment of insulin resistance (HOMA-IR) added independent prognostic value in patients without myocardial perfusion defects. In a multivariable analysis, HOMA-IR was inversely associated with low RHI. Furthermore, elevated HOMA-IR was associated with decreased levels of vascular endothelial growth factor D, stem cell factor and endocan as well as to increased level of interleukin-6. Global gene expression pathway analysis of whole blood cells showed that high HOMA-IR and impaired endothelial function were associated with upregulated pro-inflammatory pathways and down-regulated eukaryotic initiation factor-2 pathway.

**Conclusions:**

Insulin resistance measured by HOMA-IR is associated with endothelial dysfunction and confers independent prognostic information in non-diabetic patients with chest pain without myocardial perfusion defects. Increased systemic pro-inflammatory state and decreased levels of pro-angiogenic vascular growth factors may be important underlying molecular mechanisms.

## Background

Myocardial ischemia has been shown to confer strong prognostic values in various patient populations [1, 2]. Traditionally obstructive, i.e., flow limiting coronary artery atherosclerosis has been in focus for coronary artery disease (CAD) management [3, 4]. However, the importance of non-obstructive CAD has gained increasing attention since approximately 40–50 % of patients with stable angina have normal or near normal coronary arteries on coronary angiography [5, 6]. Despite the lack of obvious obstructive CAD, many of these patients are still at increased risk for future cardiovascular events [5, 7]. Indeed, a recent study revealed high prevalence of microvascular dysfunction in patients with chest pain and non-obstructive CAD [8].

Stress myocardial perfusion scintigram (MPS) is of strong prognostic value identifying hemodynamically significant obstructive CAD in patients with suspected myocardial ischemia [2]. A recent study suggests MPS to better assess functionally significant CAD compared to coronary angiography in patients with intermediate pre-test probability [9]. However, since MPS provides relative flow distribution pattern rather than absolute flow, it might still be less sensitive to detect coronary microvascular disease, including endothelial-dependent as well as independent coronary microvascular abnormalities in patients with chest pain and non-obstructive CAD [10]. Recently, we reported a role of assessing peripheral endothelial function in risk stratification of patients with chest pain without myocardial perfusion defects. This further indicates that other pathophysiological features than hemodynamically significant macrovascular disease may be responsible for clinical outcome of patients with angina-like symptoms [11].

Endothelial dysfunction is a consequence of different mechanisms causing impaired vasodilation or increased vasoconstriction [12, 13] leading to microvascular dysfunction [14]. In addition to endothelial dysfunction, the involvement of vascular remodeling, rarefaction and collaterals are important mechanisms [13], emphasizing its complexity. Microvascular dysfunction in the absence of obstructive CAD may be an early sign of cardiovascular risk. Consequently, understanding of associated mechanisms could be of great importance in management of cardiometabolic diseases. Diabetic patients are known to be at high risk for development of premature cardiovascular complications [15]. In fact, type 2 diabetes diagnosis is preceded by years of impaired glucose homeostasis, which may contribute to early pathological alteration in the cardiovascular system. Pre-diabetes may cause functional [16, 17] as well as potentially structural vascular changes [18, 19], eventually leading to clinical manifestation of the underlying vascular diseases.

Cardiometabolic diseases may involve a complex chain of events including visceral adiposity, metabolic syndrome, Type 2 diabetes and CAD. In the present work we hypothesized that insulin resistance, assessed by homeostasis model assessment of insulin resistance (HOMA-IR), in non-diabetic patients is associated with peripheral endothelial dysfunction and worse clinical outcome in patients with chest pain without myocardial perfusion defects. Further, for deepened mechanistic understanding, we explored plasma protein and gene expression patterns in whole blood cells associated with HOMA-IR.

## Methods

### Subjects and study design

**Fig. 1:**
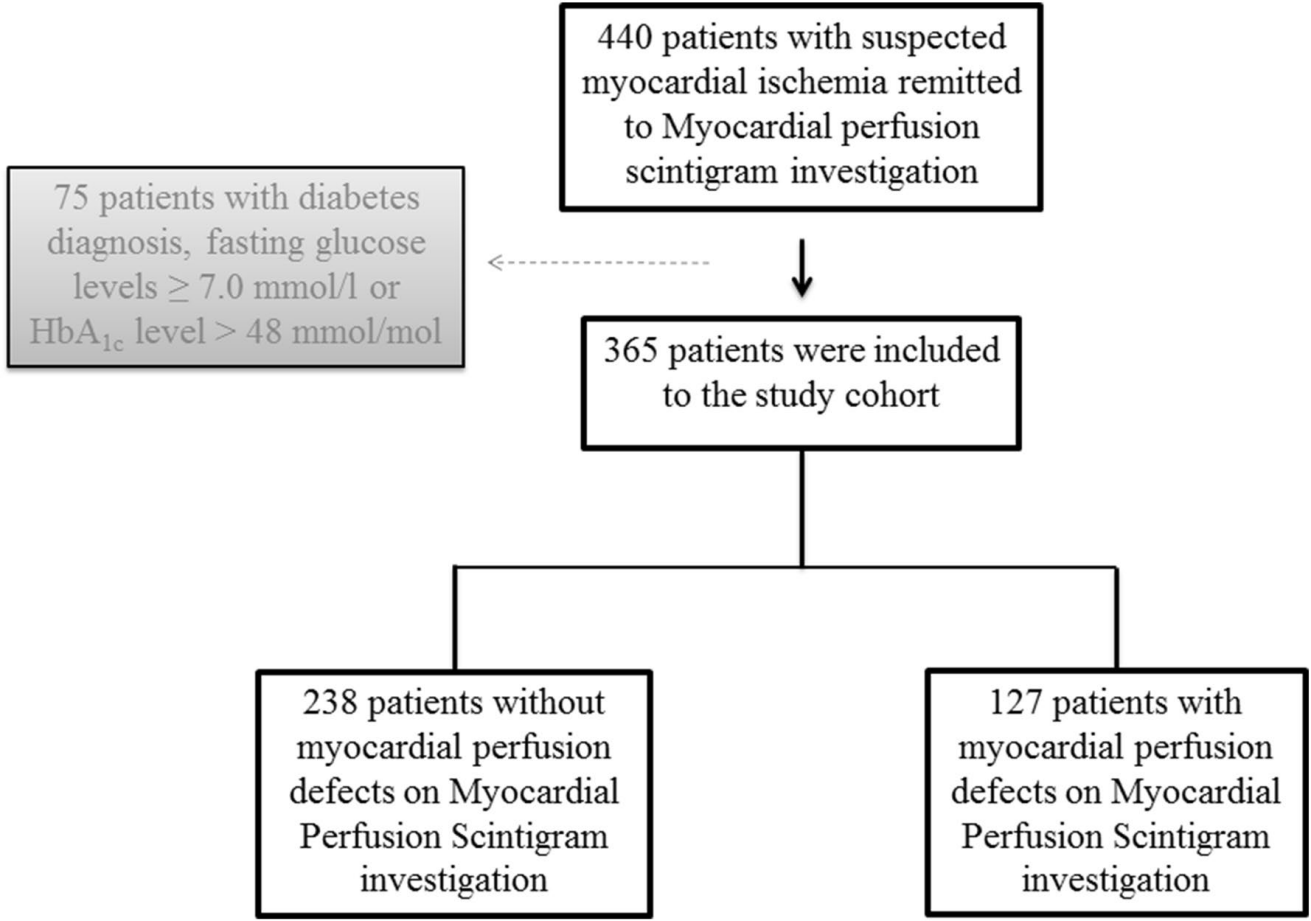
A flow-scheme illustrating the patient recruitment process

A total of 365 consecutive non-diabetic patients with chest pain referred to Sahlgrenska University Hospital in Gothenburg, Sweden due to suspected myocardial ischemia, were recruited to the study between 2006 and 2008 (Fig. 1). At a separate occasion within 2 weeks following clinical MPS examination, all patients underwent examinations of peripheral endothelial function. Overnight fasting blood samples were taken post examination of endothelial function. All patients underwent a standardized interview of medical history including history of diabetes, known CAD, smoking status and current cardiovascular medication. Patients with previous diabetes diagnosis, fasting plasma glucose ≥7.0 mmol/L or glycated haemoglobin A1c (HbA_1c_) level >48 mmol/mol were excluded from the study. Known CAD was defined as previous coronary artery bypass grafting, percutaneous coronary intervention or myocardial infarction (MI) and was collected from patients’ medical records. All participants provided written informed consent. The study complies with the declaration of Helsinki and was approved by the Local Ethics Committee at the University of Gothenburg.

### Myocardial perfusion scintigram examination

Gated-SPECT studies were performed with a 2-day standard clinical protocol using ^99m^Tc-sestamibi. Images were obtained using dual-head SPECT cameras (Infinia or Millennium VG, GE Healthcare, Milwaukee, Wisconsin, USA). The severity of reversible myocardial ischemia was scored as no (score 0), mild (score 1), moderate (2) or severe (3). The extent was scored as none, small, medium or large (score 0 for no sign of ischemia, score 1 for <10 %, score 2 for 10–19 %, score 3 for >19 % of the entire myocardium being affected, respectively). No myocardial perfusion defects was defined when both extent and severity of ischemia were scored 0.

### Endothelial function measured by reactive hyperemic index

To determine peripheral endothelial function in this patient group we performed peripheral arterial tonometry. The technician performing the examination was blinded to all clinical patient data including MPS results. Measurements were recorded using the EndoPAT2000 device (Itamar Medical, Caesarea, Israel). Endothelial function as assessed by reactive hyperemic index (RHI) has previously been described [20]. Briefly, a designed probe is placed bilaterally on each index finger. Following automatic expansion of the finger probe cuff, distal digit volume changes are calculated from pressure alterations through pressure transducers connected to the EndoPAT2000 device. RHI is calculated automatically as the ratio between the generated signals 5-min post forearm occlusion and at baseline, in relationship to the response in the contralateral arm.

### Laboratory analyses

Blood samples were collected after an overnight fast. The biochemical analyses were performed using commercially available kits according to the manufacturers’ protocols; serum triglycerides and total cholesterol (Roche Diagnostics GMBH, Mannheim, Germany), direct high density lipoprotein (HDL) cholesterol (Horiba ABX, France), Apolipoprotein A1, Apolipoprotein B (DakoCytomation, Glostrup, Denmark) and serum insulin (Millipore Corporation, USA). Plasma glucose was measured using a photometric method and HbA_1c_ by HPLC at the department of Clinical Chemistry, Sahlgrenska University Hospital, Gothenburg. Impaired glucose homeostasis, assessed by HOMA-IR was calculated to estimate insulin resistance using the formula: fasting serum insulin (mU/L) × fasting plasma glucose (mmol/L)/22.5 [21]. A number of cardiovascular associated biomarkers (stem cell factor, vascular endothelial growth factor D (VEGF-D), vascular endothelial growth factor A (VEGF-A), interleukin-6 (IL-6), and endothelial cell-specific molecule 1 also known as endocan were analyzed using the Olink Bioscience (Uppsala, Sweden) Proseek Multiplex CVD I 96 × 96 according to the manufacturer’s instructions [22]. All Proseek data are presented as arbitrary units (AU) in log^2^ values.

### Follow-up and definitions of outcome measures

Follow-up was accomplished by a physician by telephone interviews and confirmed through patient’s medical record and/or the Swedish National Board of Health’s Registry and median follow-up time was 5.1 years (range: 4.4–6.2 years). Study endpoint was event-free survival and events were defined as the incidence of all-cause mortality, nonfatal stroke, nonfatal MI and coronary arterial revascularizations (either coronary artery bypass grafting or percutaneous coronary intervention). Time to most severe event was analyzed and ranged as follows death > MI/stroke > arterial revascularization. Definition of MI was clinically diagnosed by presence of persistent chest pain and confirmed by pathological troponin dynamics and/or electrocardiogram changes. The occurrence of stroke was defined as focal or global neurological deficits lasting for more than 24 h and verified clinically by a neurologist and/or by computed tomography brain scan.

### Samples and gene expression analysis on patients without myocardial perfusion defects

Extraction of RNA was done on whole blood samples collected using Paxgene tubes from a subset of 54 consecutive patients without myocardial perfusion defects, in conjunction to the imaging procedure. Affymetrix Gene Arrays (Human Gene 1.0 ST array) was used to interrogate 28,869 transcript clusters and CEL files were imported to Partek Genomics Suite version 6.5 (Partek Inc., MO, USA). The data was log_2_-transformed and Robust Multi-array Average normalization was performed using ArrayStudio (Omicsoft, version 3.2.0 and 3.5.0). The normalized gene expression values were then fitted with a linear regression model using the Bioconductor R package ‘limma’ (linear models for microarray data) version 3.22.7. Using an empirical Bayesian approach, the ‘limma’ package infers differential expression in individual genes from the microarray data [23].

### Partial least squares—discriminant analysis, functional and pathway analysis

The application of partial least squares—discriminant analysis (PLS-DA) to the microarray data of the 54 patient samples was carried out using the MATLAB^®^ software v. 8.3.0.532 (R2012a, The MathWorks, Inc.) and the MATLAB^®^ PLS Toolbox v. 7.5.2 (Eigenvector Research, Inc.). PLS-DA scores and weights plot were used to identify markers, from the ‘limma’ gene expression analysis results, correlated to patients with high HOMA-IR (cut-off value of median of HOMA-IR >3.1); and correlated to patients with decreased endothelial function (low RHI based on RHI median of <1.91). The PLS-DA scores plot based on the cut-offs for HOMA-IR and RHI levels showed discrimination of the patient samples into two classes; and the weights plot for the appropriate latent variables revealed which genes were important in separating the classes and hence association to the appropriate class (data not shown).

To define genes associated to high HOMA-IR, and low RHI; and identify the biological mechanisms, pathways and functions co-regulated by the genes, core analyses were performed using the ingenuity pathway analysis (IPA) software (Ingenuity Systems, Redwood City, CA). The significance of the connection between the expression data and the canonical pathway were calculated by ratio and/or Fisher’s exact test. Significant genes passing the test criterion (e.g., *t* test, correlation analysis p-value for ANOVA or fold change) were functionally categorized by gene ontology [24]. This resulted into associated genetic networks, canonical pathways, and biological functions enriched by the genes. Comparison analysis in IPA for the core analysis results was then carried out to identify co-regulated pathways.

### Statistics

Deviations in sample size for the various statistical analyses were due to differences in the availability of RHI, as well as missing values in some analyzed biomarkers. All analyses were performed in SPSS, (version 21.0, Chicago Inc, USA). P-values of less than 0.05 were considered significant (2-tailed). Due to there is no present “golden standard” cut of value for HOMA-IR, we used the median value. We calculated the sample size based on the Cox PH one-sided superiority formula. With an overall event rate 20 %, an alpha level 5 and 80 % power, we need approximately 260 patients to estimate a hazard ratio (HR) of 2. Sub-analyses were performed in patients with and without myocardial perfusion defects, defined as above. Values are displayed as mean ± SD for continuous variables and frequency and percentages for categorical variables. The test of skewness was used to assess normal distribution. Non-normally distributed variables are presented with their median and interquartile range. Differences among continuous variables were analyzed using unpaired t test or Mann–Whitney U test, as appropriate. Categorical data was analyzed by Pearson Chi square test. Spearman’s correlation coefficients were used to examine relationships between continuous variables. Continuous and categorical HOMA-IR, divided by the median value was used in a linear regression model, predicting continuous RHI in patients without myocardial perfusion defects. Possible co-linearity between the independent variables was tested using Spearman correlation coefficient test and a coefficient >0.7 was considered significant. High co-linearity was found between fasting glucose levels and pre-diabetes diagnosis (correlation coefficient 0.8). The latter was added to the multivariable model investigating factors of importance for outcome. The multivariable linear regression model was adjusted for categorical HOMA-IR median and other relevant independent parameters associated to the dependent parameter with a p < 0.25 (gender, previously known CAD, body mass index and HDL). Results are displayed as β-values and 95 % confidence intervals (CI). Kaplan–Meier curves are used to display survival rates. HR and 95 % CIs were analyzed by univariate Cox regression with continuous and categorical HOMA-IR, divided by the median value in the whole population as well as in patients with and without myocardial perfusion defects. Also multivariable COX regression analyses were performed with HOMA-IR as a categorical variable divided by the median value, adjusted for age, body mass index, gender, systolic blood pressure, HDL and previously known CAD, selected based on their observed relevance to the dependent parameter (univariate Cox regression, p < 0.25) in the current study.

## Results

### Demographic and clinical characteristics

Demographic data of the 365 non-diabetic patients with suspected myocardial ischemia are shown in Table 1. HbA_1c_ levels were normally distributed within the range 4.3-6.5 % (24–48 mmol/mol), of which 4 subjects are above the 95:th percentile, normal range 4.6–6.4 % (27–46 mmol/mol) as by local definition (Department of Clinical Chemistry, Sahlgrenska University Hospital, Gothenburg, Sweden). Fasting serum insulin levels showed a right skewed distribution with the range 28–493 pmol/L. Fasting plasma glucose levels were normally distributed with the range 4.0–6.8 mmol/L. Also HOMA-IR showed a right skewed distribution with the range 1.1–14.0. Among the 365 patients, 116 subjects (32 %) were found to have impaired fasting glucose levels (100–125 mg/dL; 5.6–6.9 mmol/L) as defined by American Diabetes Association. Impaired fasting glucose levels (≥5.6 mmol/L) was used to define patients with pre-diabetes in subsequent analyses.

**Table 1:** Baseline characteristics of study cohort

	Whole study population (n = 365)	Without perfusion defects (n = 238)
Age (years)	62 ± 9	61 ± 9
Women	202 (55 %)	150 (63 %)
Body mass index	25.7 ± 3.5	25.4 ± 3.5
Current smoker	47 (13 %)	31 (13 %)
Family history of CAD	142 (39 %)	89 (37 %)
ACE-inhibitors	69 (19 %)	32 (13 %)
Beta-blockers	173 (47 %)	96 (40 %)
Statins	146 (40 %)	83 (35 %)
Aspirin	178 (49 %)	102 (43 %)
Systolic blood pressure (mmHg)	144 ± 23	144 ± 22
Known CAD	94 (26 %)	44 (18 %)
Previous MI	51 (14 %)	19 (8 %)
Triglycerides (mmol/L) (n = 362)	1.2 (0.8–1.6)	1.1 (0.8–1.3) (n = 235)
Cholesterol (mmol/L) (n = 360)	5.4 ± 1.3	5.4 ± 1.1 (n = 234)
HDL (mmol/L)	1.47 (1.24–1.72)	1.49 (1.29–1.75)
ApoB/ApoA1	0.64 (0.53–0.79)	0.62 (0.52–0.76)
Fasting glucose (mmol/L)	5.3 ± 0.5	5.3 ± 0.5
Insulin (pmol/L)	94 (69–125)	92 (69–122)
HbA_1c_ (mmol/mol, %)	36.6 ± 3.8 (5.5 ± 0.3 %)	36.2 ± 3.5 (5.5 ± 0.32 %)
HOMA-IR	3.1 (2.3–4.3)	3.1 (2.3–4.1)
RHI (n = 345)	1.89 (1.55–2.47)	1.91 (1.59–2.48) (n = 225)

Values are displayed as mean ± SD or median and interquartile range for continuous variables and frequency and percentages for categorical variables

*ApoA* apolipoprotein A, *ApoB* apolipoprotein B, *HbA*_*1c*_ glycosylated hemoglobin, *HDL* high density lipoprotein cholesterol. *MI* myocardial infarction, *Known CAD* previously known coronary artery disease, *RHI* reactive hyperemic index

### HOMA-IR as predictor of clinical outcome

**Fig. 2:**
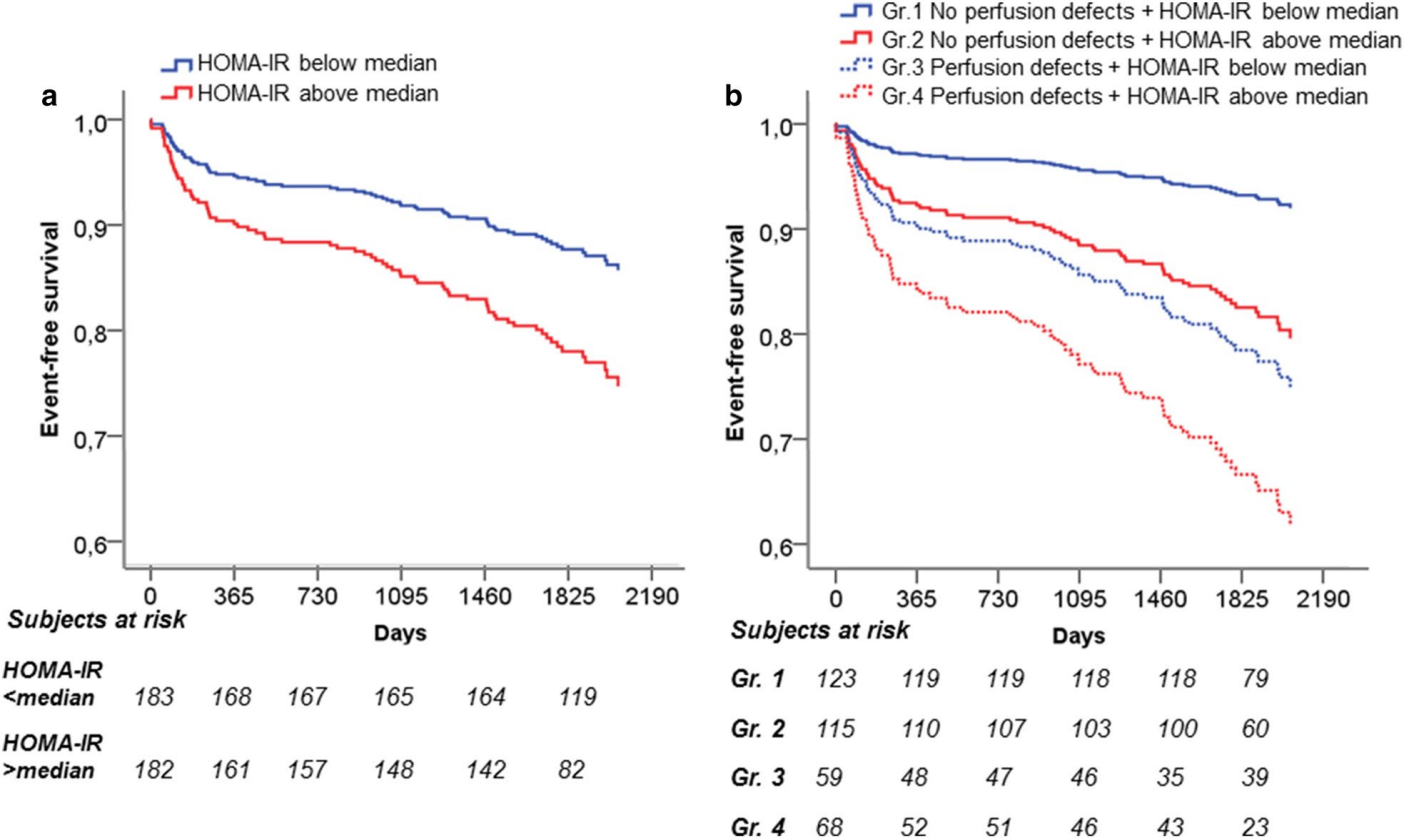
COX regression analyses between HOMA-IR below and above median on non-diabetic patients with suspected myocardial ischemia (n = 365). In a COX regression analysis **a** HOMA-IR above median provides an independent prognostic value predicting long-term events in the whole study population. In a COX regression analysis **b** HOMA-IR provides independent prognostic information in non-diabetic patients with no myocardial perfusion defects (HR: 2.7, p = 0.02, n = 238), but not in patients with myocardial perfusion defects (HR: 1.7, p = 0.14, n = 127). Statistics are presented as Chi square values and HR. *Gr* group, *HR* hazard ratio

The median follow-up time in our study was 62 months (5.1 years) with a range of 53–74 months. There was no loss to follow-up. The occurrence of events during the follow-up time in the study population was 21 % (death n = 15, MI/stroke n = 15, coronary revascularization n = 52). In a univariate analysis continuous HOMA-IR (HR: 1.13, CI: 1.04–1.24, p = 0.005, χ^2^: 7.9) predicted event-free survival. Furthermore, in a univariate survival analysis (Table 2) HOMA-IR (χ^2^: 17.1) above median value significantly predicted outcome together with age (χ^2^: 15.3), previously known CAD (χ^2^: 30.6), gender (χ^2^: 23.6), HDL (χ^2^: 7.7) and systolic blood pressure (χ^2^: 4.4). In a multivariable model of survival analysis (Table 2) adjusting for relevant risk factors (p < 0.001, χ^2^: 55.0), HOMA-IR above median independently predicted outcome (Fig. 2a) together with age, previously known CAD and gender. Also when adding pre-diabetes (univariate survival analysis: HR: 1.7, CI: 1.1–2.7, p = 0.020, χ^2^: 5.5) to the multivariable model, HOMA-IR above median (HR: 1.8, CI: 1.1–3.2, p = 0.042, χ^2^: 55.1) but not pre-diabetes (HR: 1.05, CI: 1.7, p = 0.854, χ^2^: 55.1) remained an independent predictor of outcome.

**Table 2:** Univariate and multivariable adjustment of HOMA-IR as predictor of clinical outcome

	Univariate model		Multivariable model	
	HR (95 % CI)	p value	HR (95 % CI)	p value
Age	1.05 (1.03–1.08)	<0.001	1.04 (1.01–1.07)	0.007
Known CAD	3.27 (2.10–5.10)	<0.001	1.87 (1.15–3.05)	0.012
Gender	3.10 (1.92–5.01)	<0.001	1.92 (1.11–3.35)	0.020
HOMA-IR above median	2.72 (1.70–4.46)	<0.001	1.88 (1.09–3.26)	0.023
HDL	0.38 (0.19–0.75)	0.005	0.82 (0.41–1.63)	0.570
Systolic blood pressure	1.01 (1.00–1.02)	0.036	1.00 (0.99–1.01)	0.693
Body mass index	1.05 (0.99–1.12)	0.120	1.00 (0.92–1.08)	0.915

Survival analyses on the whole study population of non-diabetic patients with suspected myocardial ischemia. Data are presented with hazard ratio and 95 % CI, (n = 365)

*CI* confidence interval, *HDL* high density lipoprotein cholesterol, *HR* hazard ratio *Known CAD* previously known coronary artery disease

### Independent prognostic value of HOMA-IR in patients without myocardial perfusion defects

Impaired glucose homeostasis in the non-diabetic state may be of importance in progression of microvascular dysfunction, i.e., in patients with chest pain without obstructive CAD. We therefore investigated the additive value of HOMA-IR to MPS results. In our cohort, the 238 patients without myocardial perfusion defects had an event rate of 13 %, while 127 patients displayed myocardial perfusion defects with an event rate of 35 %. In patients without myocardial perfusion defects the difference in event rate in patients with HOMA-IR below versus above median was 15 % (p = 0.001), and in patients with myocardial perfusion defects, the difference was 22 % (p = 0.008). Furthermore, HOMA-IR showed additive value for outcome when combined with MPS results. High versus low HOMA-IR had significant prognostic value in patients without (HR: 3.4, CI: 1.5–7.5, p = 0.003, χ^2^: 45.0) and with myocardial perfusion defects (HR: 2.1, CI: 1.4–4.0, p = 0.018, χ^2^: 45.0). In the multivariable model including conventional risk factors (age, gender, systolic blood pressure, body mass index, HDL and previously known CAD (p < 0.001, χ^2^: 71.1), HOMA-IR showed independent prognostic value in patients without myocardial perfusion defects (HR: 2.7, CI: 1.2–6.3, p = 0.02), but not in patients with myocardial perfusion defects (HR: 1.7, CI: 0.3–1.2, p = 0.14) (Fig. 2b). Importantly, HOMA-IR above median remained an independent predictor of outcome in patients without myocardial perfusion defects also when adding pre-diabetes (HOMA-IR HR: 2.7 CI: 1.1–6.3, p = 0.02) to the multivariable model (χ^2^: 71.2). Moreover, when using the first multivariable model and excluding the 94 patients with previously known CAD, HOMA-IR above median increasingly predicted outcome in patients without myocardial perfusion defects (HR: 5.0, CI: 1.4–18.3, p = 0.014) alongside age (HR: 1.05, CI: 1.01–1.09, p = 0.008). However, HOMA-IR had no independent prognostic value in patients with myocardial perfusion defects (HR: 1.5, CI: 0.59–3.9, p = 0.388) when patients with previously known CAD were excluded.

### HOMA-IR is associated with decreased peripheral endothelial function in patients without myocardial perfusion defects

Insulin resistance and endothelial dysfunction are known to coexist [14]. Therefore, we investigated the association of HOMA-IR to peripheral endothelial function in non-diabetic patients with chest pain without myocardial perfusion defects. Continuous HOMA-IR displayed a significant negative association with RHI (β: −0.014, (CI: −0.023–0.004), p = 0.004). Also, patients with HOMA-IR in the upper compared to lower median value displayed a significant inverse association with RHI (Table 3) and we observed a significantly higher RHI value in patients above compared to below median value (2.2 ± 0.7 and 1.9 ± 0.7, respectively, p = 0.001). Additionally, in a multivariable linear regression analysis (Table 3) adjusting for risk factors associated with RHI in our study, HOMA-IR above median independently predicted decreased RHI (R square = 0.05).

**Table 3:** Univariate and multivariable parameters associated to reactive hyperemic index in patients without myocardial perfusion defects

	Univariate		Multivariable model	
	β (95 % CI)	p	β (95 % CI)	p
HOMA-IR above median	−0.273 (−0.443–0.102)	0.002	−0.230 (−0.426–0.034)	0.022
Body mass index	−0.020 (−0.046–0.006)	0.129	−0.009 (−0.038–0.020)	0.548
HDL	0.160 (−0.068–0.388)	0.167	0.079 (−0.176–0.335)	0.541
Gender	0.124 (−0.057–0.305)	0.177	−0.028 (−0.241–0.185)	0.795
Known CAD	0.139 (−0.083–0.362)	0.219	0.091 (−0.143–0.324)	0.444

Linear regression analyses on non-diabetic patients without myocardial perfusion defects (n = 225)

*CI* confidence interval, *HDL* high density lipoprotein cholesterol, *Known CAD* previously known coronary artery disease

### HOMA-IR and cardiovascular biomarkers in patients without myocardial perfusion defects

Furthermore, we aimed to investigate the relation of increased HOMA-IR to inflammatory and vascular growth factor associated biomarkers in patients without myocardial perfusion defects (n = 238). The systemic low degree inflammation marker IL-6 showed positive correlation to HOMA-IR (corr.coeff = 0.200, p = 0.002) and a significantly higher value in patients with HOMA-IR above compared to below median value (3.8 ± 1.1 and 3.4 ± 0.9 AU, respectively, p = 0.008). The growth factor VEGF-D was inversely correlated to HOMA-IR (corr.coeff = −0.213, p = 0.001) and was significantly lower in patients with HOMA-IR above compared to lower median value (6.3 ± 0.3 and 6.1 ± 0.4 AU, below versus above median, respectively, p = 0.04). Reduced levels of VEGF related factor endocan also called the endothelial cell-specific molecule 1 was further associated with high HOMA-IR (corr.coeff = −0.249, p < 0.001) and lower in patients with HOMA-IR above median (2.5 ± 0.3 and 2.3 ± 0.3 AU, below versus above median, respectively, p = 0.006). We observed positive correlation between VEGF-D and endocan (corr.coeff = 0.375, p < 0.001), which confirmed the relationship suggested in previous studies [25]. However, no significant correlation was found between VEGF-A and HOMA-IR (corr.coeff = 0.062, p = 0.34) and no difference was found between VEGF-A in patients with HOMA-IR above compared to below median (9.0 ± 0.4 and 9.0 ± 0.3, respectively). Furthermore, high HOMA-IR was inversely correlated with Stem cell factor (corr.coeff = −0.219, p = 0.001) and patients with HOMA-IR above median had reduced level of Stem cell factor (8.1 ± 0.4 and 8.0 ± 0.3 AU, below versus above median, respectively, p = 0.001).

### Gene Expression, PLS-DA and pathway analyses in patients with high HOMA-IR and low RHI

**Fig. 3:**
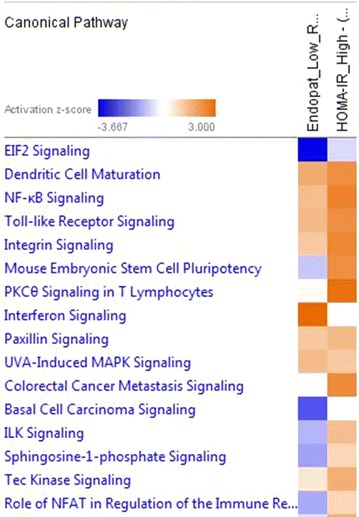
Global gene expression pathway analysis on patients without myocardial perfusion defects (n = 54). *Red bars* predict an overall increase in the activity of the pathway (activation) while *blue bars* indicate a prediction of an overall decrease in activity (deactivation/inhibition). *White bars* are those with a z-score which is zero or very close to zero. The overall activation/inhibition (deactivation) states of the pathways are predicted based on a Z-score algorithm. Z-score gives a statistical measure of the relationship of up and downregulated gene transcripts in the microarray data set with-respect-to a particular pathway. This Z-score is used to mathematically compare the microarray data set with the canonical pathway patterns. A pathway is predicted as activated or inhibited by comparing the expected pattern (up/downregulation of key genes in the pathway) if the pathway is activated against the actual pattern (up/downregulated key genes) in the microarray data set. If the actual pattern matches the expected pattern, the Z-score is positive (Z-score > ~2 = activated pathway) otherwise negative (Z-score <2 = inhibited pathway)

The outcome of ‘limma’ gene expression analysis is moderated t-statistics with Bayesian-adjusted denominators that incorporate information across all genes [26]. The ‘limma’ result consisting of the probeset IDs and the log-fold expression values of gene transcripts were then analyzed using the IPA software. Differential expressed genes were characterized having an estimated fold-change >1.5, and the Benjamini and Hochberg’s method was used to control false discovery rate smaller than 0.05 [27]. Further analysis by PLS-DA produced lists of gene transcripts associated to HOMA-IR above median and RHI below median. Thereafter, we examined the canonical pathways enriched. IPA comparison analysis gene transcripts predict patients with high HOMA-IR and low RHI co-regulated signaling pathways. Worth mentioning is the activated pro-inflammatory pathways, dendritic cell maturation signaling, nuclear factor kappa B signaling and toll-like receptor signaling as well as inhibited eukaryotic initiation factor 2 (EIF2) signaling (Fig. 3).

## Discussion

We show how elevated HOMA-IR, a marker of insulin resistance [28], independently predicts outcome in non-diabetic patients with chest pain symptoms and suspected myocardial ischemia. Importantly, HOMA-IR has independent prognostic value in patients without myocardial perfusion defects and in this subgroup increased HOMA-IR was associated with decreased endothelial function. Furthermore, in patients without myocardial perfusion defects increased HOMA-IR was associated with increased circulating levels of IL-6 and decreased levels of vascular growth factors, suggesting systemic inflammation and impaired angiogenic potential. In support to the protein level data, global gene expression analysis revealed that co-regulated pro-inflammatory pathways and down-regulated EIF2 signaling pathway were associated with high HOMA-IR and impaired endothelial function.

### HOMA-IR as predictor of clinical outcome

MPS is a well-established tool in detecting hemodynamically significant obstructive CAD and has strong prognostic value [2]. However MPS may not be the optimal modality to detect global microvascular dysfunction, which typically requires absolute flow reserve quantification [29]. In the current non-diabetic population, increased insulin resistance as measured by HOMA-IR predicted future events. Interestingly, an independent prognostic value of HOMA-IR was only observed in patients without myocardial perfusion defects. In line with previous reports, patients with perfusion defects may already be at high risk for cardiovascular events, so testing HOMA-IR may not add independent values for prognostic evaluation [11]. The fact that an independent prognostic value of HOMA-IR was observed in non-diabetic patients without myocardial perfusion defects independently of pre-diabetes, supported our hypothesis that insulin resistance could be one of the main mechanisms associated with increased cardiovascular risk in these patients. Further, assessing HOMA-IR may add clinical value also in patients with long standing type 2 diabetes. Srinivasan et al. recently showed that HOMA-IR below 2.5 is associated with normal coronary angiogram indicating importance of assessing insulin resistance also to help to predict outcome in type 2 diabetes patients [30].

### HOMA-IR and peripheral endothelial function

The results of the present study indicate that insulin resistance is associated with peripheral endothelial dysfunction and predicts clinical outcome also in patients without myocardial perfusion defects. It was recently shown that the prevalence of microvascular dysfunction in patients with chest pain and non-obstructive CAD is high in both men and women and that conventional risk factors play a minor role [8]. In the present study, endothelial function was assessed by Endo-PAT, which has been shown to predict coronary endothelial dysfunction [20] as well as having additive prognostic value to the Framingham risk score [11]. HOMA-IR is a surrogate measure of insulin resistance that has been shown to be more strongly associated with cardiovascular disease than glucose or insulin concentrations alone in non-diabetic patients [31]. Endothelial dysfunction is a consequence of different mechanisms associated with insulin resistance causing impaired vasodilation or increased vasoconstriction [12–14]. Normal insulin action results in vasodilation at the arterial, venous, and microcirculatory levels [32] via increased production of endothelial nitric oxide [14, 17, 32]. In accordance, insulin resistance has been shown to reduce the expression and function of the endothelial nitric oxide-synthase gene in endothelial cells and microvessels in insulin resistant rats [16]. Interestingly, insulin resistance in apparently healthy adolescents is associated with endothelial dysfunction [33]. Furthermore, microalbuminuria is known to correlate with endothelial dysfunction and has been independently associated with insulin resistance in type 2 diabetes and it was recently shown that HOMA-IR below 2.5 and absence of microalbuminuria is associated with beneficial CAD profile [30].

### HOMA-IR and angiogenic factors

The pathophysiological mechanisms underlying microvascular dysfunction is multifactorial [13]. In addition to e.g., endothelial dysfunction, structural vascular adaption, including vascular remodeling, rarefaction, and collateralization could also play roles [13]. Ischemia is known to stimulate angiogenesis, and conditions of only minimal ischemia stimulate development of collateral vessels [34]. The growth factor Stem cell factor is important in mobilization and recruitment of vascular angiogenic endothelial progenitor cells in response to ischemia [35]. Interestingly, decreased Stem cell factor levels were recently found in diabetic patients and were related to incidence of cardiovascular events [19]. In our study, decreased Stem cell factor correlated significantly to high HOMA-IR and decreased VEGF-D levels (data not shown). VEGF-D has been shown to be important for vascular angiogenesis and lymphangiogenesis, as well as stimulating endothelial production of nitric oxide [36, 37]. In the current study increased HOMA-IR was inversely correlated to VEGF-D at a protein level. We further demonstrate that VEGF-D correlates positively to endocan, which is known to be induced by VEGF and involved in tumor angiogenesis [25]. In line with our data, He et al. showed that reduced PI3K/Akt signaling is likely responsible for the reduction in VEGF induced vascularization in the myocardium at both basal and ischemic states [18]. Furthermore, Bonner et al. showed that deletion of VEGF in murine cardiac muscle induced capillary rarefaction and promoted insulin resistance [38]. Taken together, these studies indicate that insulin resistance might promote downregulation of VEGF, causing vascular rarefaction which may accelerate further development of insulin resistance due to insufficient delivery of insulin. Ischemia-induced hypoxia is also a trigger for increased transcription and translation of vascular endothelial growth factors [34] ultimately leading to angiogenesis. The current study displayed impaired EIF2 pathway to be associated with insulin resistance and endothelial dysfunction. Since VEGF mRNA translation is dependent on the EIF2 pathway, especially during hypoxia [39], an impaired EIF2 pathway could at least in part be responsible for the low VEGF-D protein levels associated with high HOMA-IR. In addition, it was recently reported a novel VEGF signaling mechanism involving the EIF2 pathway to activate the unfolded protein response in endothelial cells [40], which seems to be important for angiogenesis and cell survival. Thus, a down-regulated EIF2 pathway may further diminish the action of VEGF on the vascular wall. Taken together, the reduced levels of VEGF-D and Stem cell factor could be mechanisms underlying endothelial dysfunction associated with insulin resistance in this patient population.

### HOMA-IR and subclinical inflammation

A large proportion of the study population was pre-diabetes patients. The pre-diabetic state is associated with increased systemic inflammation [41] and IL-6 has been shown to be related to coronary microvascular dysfunction [42]. We demonstrated that high HOMA-IR was associated with increased levels of IL-6 also in this cohort, which is in line with previous findings that obese, non-diabetic insulin resistant patients have elevated IL-6 levels [43]. Interestingly, Antoniades et al. showed increased IL-6 levels in healthy subjects to be associated with impaired peripheral flow-mediated vasodilation and to increased levels of asymmetrical dimethylarginine, an endogenous inhibitor of endothelial nitric oxide synthase [44]. In line with these results, IL-6 was demonstrated to inhibit endothelium-dependent nitric oxide-mediated relaxation and enhance contraction in an experimental model [45], which suggests a direct role of IL-6 in microvascular dysfunction associated with insulin resistance. In accordance, gene expression and pathway analysis indicated upregulation of multiple pro-inflammatory pathways including dendritic cell maturation signaling pathway and pattern recognition receptors signaling pathway, which both could result in e.g., increased systemic levels of IL-6.

### Study limitations

Despite extensive biomarker analysis several other important markers for cardiovascular risk may still be missing, such as adiponectin [46–48] and microalbuminuria [30]. Global gene expression pathway analysis was performed on RNA extracted from whole blood cells, used as a surrogate cell compartment mirroring similar biological processes in other relevant tissues. Caution should be taken when extrapolating the finding to other target tissues, such as endothelial cells. Further, more studies are warranted to investigate mechanistic relevance in other populations. Finally, the current study is still of limited size and therefore caution should be taken when interpreting the data.

## Conclusions

Our study shows that pre-diabetes as indicated by impaired glucose homeostasis is prevalent in non-diabetic patients with chest pain and suspected myocardial ischemia. Elevated HOMA-IR is associated with decreased endothelial function, and adds independent prognostic information in patients without myocardial perfusion defects. Furthermore, our results also indicate that increased systemic low degree inflammation and decreased vascular growth factor production may be underlying mechanisms connecting insulin resistance with microvascular dysfunction in this patient population.

## Abbreviations

ApoA: apolipoprotein A
ApoB: apolipoprotein B
AU: arbitrary units
CAD: coronary artery disease
CI: confidence interval
EIF2: eukaryotic initiation factor 2
HbA_1c_: glycated haemoglobin A1c
HDL: high density lipoprotein cholesterol
HOMA-IR: homeostasis model assessment of insulin resistance
HR: hazard ratio
IL-6: interleukin-6
IPA: ingenuity pathway analysis
MI: myocardial infarction
MPS: myocardial perfusion scintigram
PLS-DA: partial least squares—discriminant analysis
RHI: reactive hyperemic index
VEGF-A: vascular endothelial growth factor A
VEGF-D: vascular endothelial growth factor D
